# Granular cell tumor of the pancreas mimicking pancreatic ductal adenocarcinoma: a case report

**DOI:** 10.1186/s12876-026-05155-z

**Published:** 2026-07-24

**Authors:** Mátyás Béndek, Raffaella Pozzi-Mucelli, Carlos Fernández Moro, Stefan Gilg

**Affiliations:** 1 Department of Clinical Pathology and Cancer Diagnostics, Karolinska University Hospital, Stockholm, Sweden; 2 Division of Pathology, Department of Laboratory Medicine, Karolinska Institutet, Stockholm, Sweden; 3 Department of Radiology, Karolinska University Hospital, Stockholm, Sweden; 4 Division of Radiology, Department of Clinical Science, Intervention and Technology, Karolinska Institutet, Stockholm, Sweden; 5 Department of Surgery, Karolinska University Hospital, Stockholm, Sweden; 6 Division of Surgery, Department of Clinical Science, Intervention and Technology, Karolinska Institutet, Stockholm, Sweden

**Keywords:** Granular cell tumor, Pancreatic ductal stricture, Pancreatic ductal adenocarcinoma, Birt-Hogg-Dubé syndrome, Case report

## Abstract

**Background:**

Granular cell tumor (GCT) is a benign Schwannian neoplasm that only exceptionally arises in the pancreas. Because of its infiltrative growth pattern, pancreatic GCT may radiologically mimic pancreatic ductal adenocarcinoma (PDAC).

**Case presentation:**

A 38-year-old woman with Birt-Hogg-Dubé syndrome underwent magnetic resonance imaging as part of surveillance and was found to have an enhancing lesion causing ductal stricture at the junction of the pancreatic corpus and cauda. Computed tomography raised a strong suspicion of pancreatic ductal adenocarcinoma. The patient underwent distal pancreatectomy.

Histologic examination revealed a 12-mm poorly circumscribed infiltrative tumor composed of sheets and nests of polygonal cells with abundant eosinophilic granular cytoplasm and small uniform nuclei. It extended along interlobular septa and encased small pancreatic ducts, providing a morphologic correlate for the ductal stricture seen on imaging. Periodic acid–Schiff with diastase (PAS-D) stain demonstrated diffuse intracytoplasmic diastase-resistant granules. Tumor cells showed diffuse expression of S100 protein, SOX10 and inhibin A, while neuroendocrine markers were negative. The findings were consistent with a granular cell tumor.

**Conclusion:**

Pancreatic granular cell tumor is a rare benign lesion that may present with imaging findings highly suspicious for PDAC. Its infiltrative growth pattern may closely mimic carcinoma on imaging and may lead to surgical resection for presumed malignancy. To our knowledge, pancreatic granular cell tumor has not previously been reported in association with Birt-Hogg-Dubé syndrome; whether this represents a coincidental finding remains uncertain.

## Introduction

Granular cell tumor (GCT) is a benign neoplasm of Schwann cell origin most commonly occurring in the skin, subcutaneous tissue, and oral cavity. Visceral involvement is rare, and primary occurrence in the pancreas is exceptional, with only a limited number of cases reported [1–5]. Because of its infiltrative growth pattern, pancreatic GCT may present radiologically as a poorly defined lesion with ductal abnormalities that are highly suspicious for pancreatic ductal adenocarcinoma.

We report a case of a pancreatic granular cell tumor detected during surveillance imaging in a patient with Birt-Hogg-Dubé syndrome, presenting with radiologic features raising suspicion of PDAC.

## Case presentation

A 38-year-old woman with known Birt-Hogg-Dubé syndrome, manifested by recurrent pneumothorax and fibrofolliculomas of the face, was referred for baseline renal surveillance with magnetic resonance imaging (MRI) because of the increased risk of renal neoplasia associated with the syndrome.

Renal MRI was performed on a 3-T MAGNETOM Vida system (Siemens Healthineers, Erlangen, Germany) and included the following sequences: (a) multi-breath-hold axial and coronal T2-weighted HASTE (Half-Fourier Acquisition Single-shot Turbo Spin-Echo) sequences with and without fat-saturation (slice thickness: 4 mm; interslice gap: 0 mm); (b) axial T1-weighted 3D gradient-echo Dixon sequences (slice thickness: 2.5 mm) before and after intravenous administration of a gadolinium-based extracellular contrast agent (gadobutrol; Gadovist^®^, Bayer, Germany), administered at a rate of 2 mL/s, followed by a bolus of 20 mL saline flush, during the arterial, venous and delayed phases (3-, 5- and 10 min delayed acquisitions); (c) coronal T1-weighted 3D gradient-echo Dixon sequences (slice thickness: 2 mm), acquired before and after contrast administration (10 min delay) and (d) free-breathing axial diffusion-weighted imaging (DWI) with b-values of 50 and 800 s/mm^2^ (slice thickness: 4.5 mm; interslice gap: 0 mm).

**Fig. 1:**
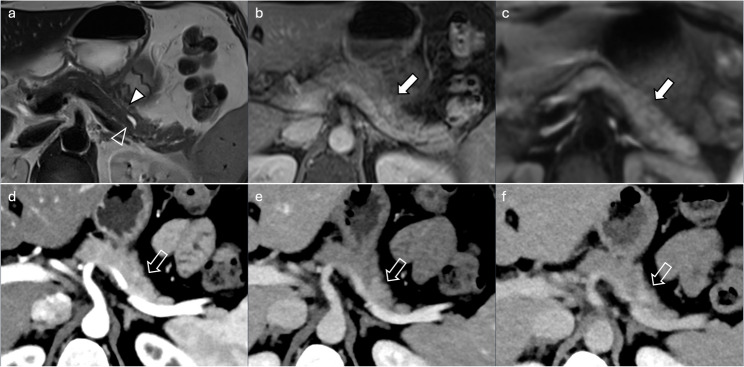
In the upper row, MR images demonstrate a short stricture of the main pancreatic duct (white arrowhead) with mild upstream ductal dilatation (open arrowhead) and modest upstream parenchymal volume loss on the axial T2-weighted image (**a**). In an axial T1-weighted fat-saturated image acquired in the delayed phase (3 min after contrast administration), an enhancing focal lesion (white arrow) is visible (**b**), showing no abnormal signal intensity on diffusion-weighted imaging (b-value 800 s/mm^2^) (**c**). In the lower row, CT images show the corresponding lesion (open arrows), appearing slightly hypodense in the pancreatic arterial phase (**d**) and mildly hyperdense in the venous (**e**) and delayed (**f**) phases

MRI demonstrated a 7-mm stricture of the main pancreatic duct at the junction of the pancreatic corpus and cauda, with focal upstream dilatation up to 3 mm (Fig. 1a). Distal to the dilated duct, an approximately 9-mm enhancing lesion was identified in the delayed phase (Fig. 1b). No abnormal signal intensity was detected on diffusion-weighted imaging (Fig. 1c). Since this was the baseline MRI examination, no prior MRI studies were available for comparison. Retrospective review of a prior abdominal computed tomography (CT) examination performed 8 years earlier for acute appendicitis did not reveal any abnormalities at the corresponding location (images not shown). Based on these findings, a small fibrotic pancreatic ductal adenocarcinoma (PDAC) or pancreatic neuroendocrine tumor was considered in the differential diagnosis, and dedicated pancreatic CT was recommended for further evaluation.

Pancreatic CT, performed 10 days after the initial MRI, revealed an approximately 2-cm poorly defined lesion in the corpus–cauda transition with slightly progressive contrast enhancement, mild upstream duct dilatation, and distal parenchymal atrophy (Fig. 1d–f), raising suspicion of PDAC. The multidisciplinary team considered that even a negative endoscopic ultrasound (EUS) would not reliably exclude malignancy. Therefore, no preoperative EUS or EUS-guided tissue sampling (EUS-FNA/FNB) was pursued, and distal pancreatectomy with splenectomy was performed.

Gross examination showed a distal pancreatectomy specimen measuring 10 cm with attached spleen and omentum. In the corpus–cauda transition, a poorly defined light-brown lesion measuring 0.8 cm was identified within the pancreatic parenchyma. The lesion appeared confined to the pancreas. The closest resection margin was posterior, at a distance of 0.7 cm. Mild upstream duct dilatation was present. The spleen and omentum were unremarkable.

**Fig. 2:**
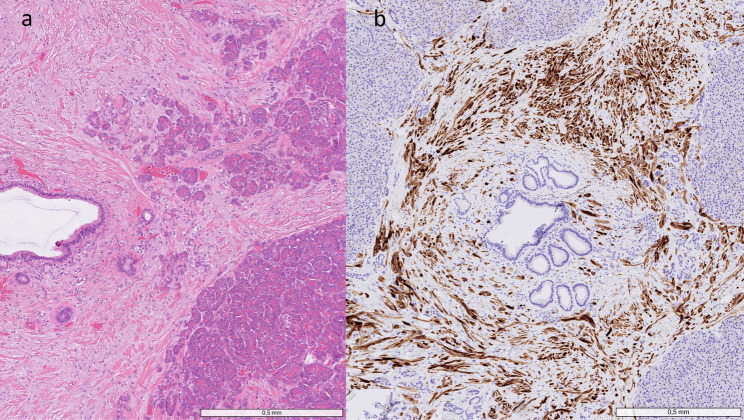
**a** Low-magnification hematoxylin–eosin stain (from whole-slide digital image), showing a poorly circumscribed infiltrative tumor extending along fibrous septa within the pancreatic parenchyma. **b** Low-magnification inhibin-α immunostain (from whole-slide digital image), highlighting tumor cells infiltrating along interlobular septa and encasing a small pancreatic duct

**Fig. 3:**
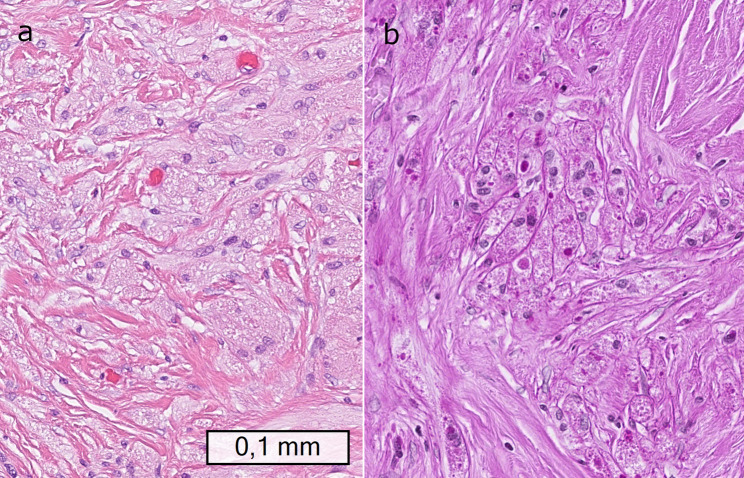
**a** High-magnification H&E stain (from whole-slide digital image) showing polygonal tumor cells with abundant eosinophilic granular cytoplasm and small uniform nuclei. **b** High-magnification periodic acid–Schiff with diastase (PAS-D) stain (from whole-slide digital image) demonstrating diffuse intracytoplasmic diastase-resistant granules characteristic of a granular cell tumor

**Fig. 4:**
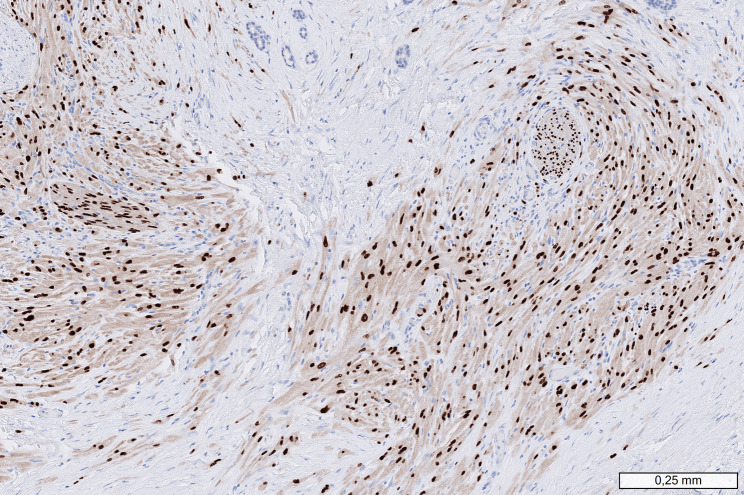
Intermediate-magnification SOX10 immunostain (from whole-slide digital image) showing diffuse nuclear positivity in tumor cells, consistent with Schwannian differentiation. Tumor cells encase small peripheral nerves, without true intraneural invasion

Histologic examination revealed a poorly circumscribed infiltrative tumor measuring 12 mm in greatest dimension, composed of sheets and nests of polygonal cells with abundant eosinophilic granular cytoplasm and small uniform nuclei without prominent nucleoli (Fig. 3a). No necrosis, increased mitotic activity or nuclear pleomorphism was seen. The tumor extended along interlobular septa and encased small pancreatic ducts (Fig. 2a, b) and also surrounded small peripheral nerves without definite intraneural invasion (Fig. 4). Periodic acid–Schiff with diastase (PAS-D) stain demonstrated diffuse intracytoplasmic diastase-resistant granules (Fig. 3b).

Immunohistochemically, the tumor cells were diffusely positive for S100, SOX10, neuron-specific enolase (NSE), CD68, inhibin-α and were negative for chromogranin A, synaptophysin, INSM1, ISLET1, desmin and HMB45 (Figs. 2b and 4). The Ki-67 index was below 1%. These findings were consistent with a granular cell tumor.

Twenty-two regional lymph nodes showed no metastases. The postoperative course was uneventful.

## Discussion

Granular cell tumor, originally described by Abrikossoff in 1926, is an uncommon benign neoplasm of Schwann cell origin that can occur in many anatomical sites, most frequently in the skin and oral cavity [1, 2]. A century after its first description, pancreatic localization of this tumor remains exceedingly rare [3–5].

Pancreatic granular cell tumors lack specific clinical or radiologic features and may present as poorly defined lesions with ductal abnormalities, frequently leading to suspicion of pancreatic ductal adenocarcinoma and surgical resection [3–5]. In the present case, imaging demonstrated a focal ductal stricture with upstream dilatation, distal atrophy and an ill-defined enhancing lesion, a constellation of findings that is considered highly suspicious for malignancy in routine clinical practice.

Histologically, granular cell tumors consist of polygonal cells with abundant lysosome-rich granular cytoplasm, show diffuse expression of S100 and other neural markers [2], and typically display an infiltrative growth pattern. In the present case, the lesion extended along interlobular septa and encased small ducts. Although direct involvement of the main duct was not demonstrated histologically, this infiltrative growth likely explains the ductal stricture seen on imaging, providing the morphologic correlate for the radiologic suspicion of pancreatic ductal adenocarcinoma.

In addition to the expected diffuse expression of S100 and SOX10, granular cell tumors frequently express inhibin-α, which has emerged as a useful adjunctive marker in challenging cases [6].

The principal histologic differential diagnosis includes acinar cell carcinoma with granular cytoplasm, oncocytic neuroendocrine tumors and, less commonly, PEComa. In the present case, the bland cytomorphology together with diffuse expression of S100, SOX10 and inhibin-α supported the diagnosis of granular cell tumor. The absence of neuroendocrine marker expression excluded an oncocytic neuroendocrine tumor, while negativity for HMB45 and desmin argued against PEComa.

The patient had Birt-Hogg-Dubé syndrome, a hereditary disorder associated with pulmonary cysts, fibrofolliculomas and an increased risk of renal neoplasia [7]. To our knowledge, pancreatic granular cell tumor has not previously been reported in this context. Given the absence of a known biological link between the folliculin pathway and Schwannian tumors, the association is most likely incidental.

Although granular cell tumor is considered benign, clinical follow-up may be appropriate because only a small number of pancreatic cases have been reported and long-term outcome data are limited.

## Conclusion

Pancreatic granular cell tumor is a rare benign neoplasm that may present with ductal abnormalities and imaging findings highly suspicious for pancreatic ductal adenocarcinoma. Its infiltrative growth pattern can closely mimic malignancy and may lead to surgical resection for suspected PDAC. Histopathological examination is required for definitive diagnosis. This case highlights an important diagnostic pitfall and represents, to our knowledge, the first reported pancreatic GCT occurring in a patient with Birt–Hogg–Dubé syndrome.

## Data Availability

All data generated or analysed during this study are included in this published article.
